# Regulation of cell proliferation by the guanosine–adenosine mechanism: role of adenosine receptors

**DOI:** 10.1002/phy2.24

**Published:** 2013-07-08

**Authors:** Edwin K Jackson, Delbert G Gillespie

**Affiliations:** Department of Pharmacology and Chemical Biology, University of Pittsburgh School of MedicinePittsburgh, Pennsylvania, 15219

**Keywords:** Adenosine, cell proliferation, guanosine, SLC19 family, SLC22A2

## Abstract

A recent study (*American Journal of Physiology* – *Cell Physiology* 304:C406–C421, 2013) suggests that extracellular guanosine increases extracellular adenosine by modifying the disposition of extracellular adenosine (“guanosine–adenosine mechanism”) and that the guanosine–adenosine mechanism is not mediated by classical adenosine transport systems (SLC28 and SLC29 families) nor by classical adenosine-metabolizing enzymes. The present investigation had two aims (1) to test the hypothesis that the “guanosine–adenosine mechanism” affects cell proliferation; and (2) to determine whether the transporters SLC19A1, SLC19A2, SLC19A3, or SLC22A2 (known to carrier guanosine analogs) might be responsible for the guanosine–adenosine mechanism. In the absence of added adenosine, guanosine had little effect on the proliferation of coronary artery vascular smooth muscle cells (vascular conduit cells) or preglomerular vascular smooth muscle cells (vascular resistance cells). However, in the presence of added adenosine (3 or 10 μmol/L), guanosine (10–100 μmol/L) decreased proliferation of both cell types, thus resulting in a highly significant (*P* < 0.000001) interaction between guanosine and adenosine on cell proliferation. The guanosine–adenosine interaction on cell proliferation was abolished by 1,3-dipropyl-8-(*p*-sulfophenyl)xanthine (adenosine receptor antagonist). Guanosine (30 μmol/L) increased extracellular levels of adenosine when adenosine (3 μmol/L) was added to the medium. This effect was not reproduced by high concentrations of methotrexate (100 μmol/L), thiamine (1000 μmol/L), chloroquine (1000 μmol/L), or acyclovir (10,000 μmol/L), archetypal substrates for SLC19A1, SLC19A2, SLC19A3, and SLC22A2, respectively; and guanosine still increased adenosine levels in the presence of these compounds. In conclusion, the guanosine–adenosine mechanism affects cell proliferation and is not mediated by SLC19A1, SLC19A2, SLC19A3, or SLC22A2.

## Introduction

Adenosine in the extracellular compartment activates multiple cell-surface receptors and thus regulates cellular function (Grenz et al. 2011). We hypothesize that extracellular guanosine is an important physiological regulator of extracellular adenosine levels and that extracellular guanosine regulates extracellular adenosine by interfering with the disposition of extracellular adenosine (Jackson et al. 2013). This concept allows for an indirect signaling role for guanosine without the need for guanosine receptors, which may (Traversa et al. 2003) or may not (Thauerer 2012) exist. Support for the concept that extracellular guanosine regulates extracellular adenosine (called the “guanosine–adenosine mechanism”) includes cellular studies showing that in the presence of added adenosine, guanosine profoundly increases extracellular adenosine levels and in proportion to the concentration of added adenosine. The best explanation for these results is that guanosine regulates the disposition of extracellular adenosine (Jackson et al. 2013).

An important unanswered question is whether the guanosine–adenosine mechanism has significant biological effects or is merely a biochemical curiosity. The present study addresses this question by examining the effects of guanosine on a cellular response reproducibly elicited by adenosine, that is, inhibition of vascular smooth muscle cell proliferation (Dubey et al. 1996a,b, 1998; Jackson et al. 2011a,b).

If the guanosine–adenosine mechanism is biologically significant, it is important to elucidate how this mechanism works. Theoretically, extracellular guanosine could alter the disposition of adenosine from the extracellular compartment by blocking processes involved in the removal of extracellular adenosine, for example, enzymes and carrier systems that metabolize and transport adenosine, respectively. However, recent studies (Jackson et al. 2013) seem to rule out the involvement of adenosine deaminase, adenosine kinase, S-adenosylhomocysteine hydrolase, guanine deaminase, equilibrative nucleoside transporters (SLC29 family members, also called ENTs), and concentrative nucleoside transporters (SLC28 family members, also called CNTs) in the interaction between extracellular guanosine and extracellular adenosine.

As studies rule out a role for the classical pathways for adenosine disposition in the guanosine–adenosine mechanism, other possibilities must be considered and explored. Along these lines, studies show that methotrexate (has a guanine-like polycyclic ring) increases extracellular adenosine levels (Chan and Cronstein 2002; Tian and Cronstein 2007) and methotrexate is transported by members of the SLC19 family of transporters (SLC19A1, SLC19A2, and SLC19A3) (Ganapathy et al. 2004). Also, SLC22A2 can mediate the transmembrane transport of guanosine analogs (Cheng et al. 2012). Therefore, we considered the possibility that these transporters might be potential actors in the guanosine–adenosine mechanism, and a second objective of this study was to test this hypothesis.

## Methods

### Chemicals

Adenosine, guanosine, 1,3-dipropyl-8-(*p*-sulfophenyl)xanthine (DPSPX), methotrexate, thiamine, chloroquine, and acyclovir were from Sigma-Aldrich (St. Louis, MO).

### Cell culture

Rat preglomerular vascular smooth muscle cells were cultured from kidneys as previously described by us (Mokkapatti et al. 1998). Kidneys were obtained from both normotensive Wistar-Kyoto rats (WKY) and spontaneously hypertensive rats (SHR) (Taconic Farms, Germantown, NY). Human coronary artery smooth muscle cells were obtained from Lonza Inc. (Allendale, NJ).

### Cell proliferation studies

[^3^H]-thymidine incorporation (index of DNA synthesis/cell proliferation) was performed to investigate the interaction of guanosine and adenosine on cell growth. Briefly, cells were plated at a density of 5 × 10^4^ cells per well in 24-well tissue culture dishes and allowed to grow to subconfluence in Dulbecco’s Modified Eagle’s Medium (DMEM)/F-12 medium containing 10% fetal calf serum (FCS) under standard tissue culture conditions. The cells were then growth arrested by feeding DMEM containing 0.4% albumin for 48 h. Growth-arrested cells were placed in DMEM containing 2.5% FCS with or without adenosine, with or without guanosine, or with or without the adenosine receptor antagonist DPSPX. After 20 h of incubation, the treatments were repeated with freshly prepared solutions but supplemented with [^3^H]-thymidine (1 μCi/mL) for an additional 4 h. The experiments were terminated by washing the cells twice with Dulbecco's phosphate-buffered saline (PBS) and twice with ice-cold trichloroacetic acid (10%). The precipitate was solubilized in 500 μL of 0.3 N NaOH and 0.1% sodium dodecyl sulfate (50°C for 2 h). Aliquots from each were placed in scintillation fluid and were counted in a liquid scintillation counter. We have previously shown in vascular smooth muscle cells that this method of assessing [^3^H]-thymidine incorporation correlates with cell number and is an accurate reflection of changes in cell proliferation (Jackson et al. 2010).

### Guanosine on adenosine levels

Experiments were performed in confluent cells (passage number between 2 and 5) in 24-well plates under standard cell culture conditions. On the day of study, cells were washed twice with 1 mL of PBS and then incubated for 1 h with 0.5 mL of PBS containing the indicated treatments. After the 1-h incubation, the medium was collected and frozen (−80°C) for later analysis of adenosine as described below.

### Analytical method for adenosine

Samples were spiked with a heavy-isotope internal standard (^13^C_10_-adenosine), and adenosine was resolved by reversed-phase liquid chromatography (Waters UPLC BEH C18 column, 1.7 μm beads; 2.1 × 100 mm; Milford, MA) and quantified using a triple quadrupole mass spectrometer (TSQ Quantum-Ultra; ThermoFisher Scientific, San Jose, CA) operating in the selected reaction monitoring mode with a heated electrospray ionization source. The mobile phase was delivered with a Waters Acquity ultra-performance liquid chromatographic system using a linear gradient involving two buffers: Buffer A, 1% acetic acid in water; Buffer B, methanol. The mobile phase flow rate was 300 μL/min. The gradient (A/B) was 0–2 min, 99.5%/0.5%; 2–3 min, to 98%/2%; 3–4 min, to 85%/15%; and 4–5 min, to 99.5%/0.5%. Sample tray temperature was set at 10°C and the column temperature was kept at 50°C. Instrument parameters were ion spray voltage, 4.0 kilovolts; ion transfer tube temperature, 350°C; source vaporization temperature, 320°C; Q2 CID gas, argon at 1.5 mTorr; sheath gas, nitrogen at 60 psi; auxillary gas, nitrogen at 55 psi; Q1/Q3 width, 0.7/0.7 units full-width half-maximum; source CID, off; scan width, 0.6 units; scan time, 0.01 sec; and collision energy, 19 volts. Mass transitions were adenosine, 268→136; and ^13^C_10_-adenosine (internal standard), 278→141. Retention time for adenosine was ∼2.37 min.

### Statistical analysis

Statistical analysis was performed with one-factor or two-factor analysis of variance (ANOVA) with post hoc comparisons using a Fisher's least significant difference (LSD) test if main effect or interaction effect *P*-values justified post hoc tests. The criterion of significance was *P* < 0.05. All values in text and figures are means and SEMs.

## Results

### Interaction between guanosine and adenosine on [^3^H]-thymidine incorporation in vascular smooth muscle cells

To examine the possibility that guanosine and adenosine interact to inhibit vascular smooth muscle cell proliferation, we examined the ability of guanosine to inhibit [^3^H]-thymidine incorporation in vascular smooth muscle cells from both conduit (coronary artery) and resistance (preglomerular) vascular smooth muscle cells. In these studies, cells were incubated with different concentrations of guanosine in the absence and presence of different concentrations of adenosine.

**Figure 1:**
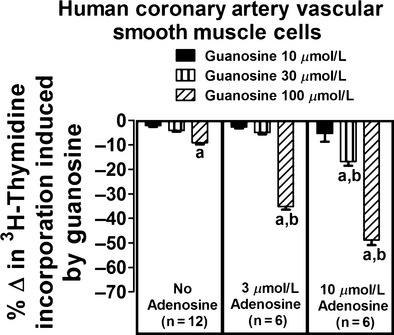
Effects of guanosine on [^3^H]-thymidine incorporation in human coronary artery vascular smooth muscle cells in the absence and presence of adenosine. Values represent means and SEMs. Two-factor ANOVA indicated a significant interaction between guanosine and adenosine on [^3^H]-thymidine incorporation (*P* < 0.000001). Letter “a” indicates a significant inhibitory response to guanosine at the indicated concentration of adenosine, and “b” indicates that the inhibitory response to guanosine is significantly greater in the presence of adenosine.

In human coronary artery vascular smooth muscle cells, in the absence of adenosine, guanosine at concentrations of 10 or 30 μmol/L had no significant effect on [^3^H]-thymidine incorporation (Fig. 1, left panel). Even at a concentration of 100 μmol/L, guanosine decreased [^3^H]-thymidine incorporation by only 9 ± 1% in the absence of adenosine. In contrast, in the presence of adenosine, guanosine markedly reduced [^3^H]-thymidine incorporation. For example, in the presence of 3 μmol/L adenosine (Fig. 1, middle panel), 100 μmol/L guanosine reduced [^3^H]-thymidine incorporation by 35 ± 1%; and in the presence of 10 μmol/L adenosine (Fig. 1, right panel), 100 μmol/L guanosine reduced [^3^H]-thymidine incorporation by 49 ± 2%. Analysis by two-factor ANOVA indicated a highly significant (*P* < 0.000001) interaction between guanosine and adenosine on [^3^H]-thymidine incorporation.

**Figure 2:**
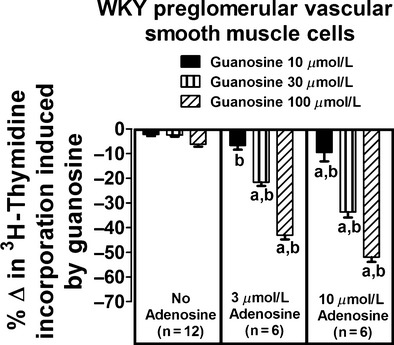
Effects of guanosine on [^3^H]-thymidine incorporation in preglomerular vascular smooth muscle cells isolated from normotensive Wistar-Kyoto rats (WKY) in the absence and presence of adenosine. Values represent means and SEMs. Two-factor ANOVA indicated a significant interaction between guanosine and adenosine on [^3^H]-thymidine incorporation (*P* < 0.000001). Letter “a” indicates a significant inhibitory response to guanosine at the indicated concentration of adenosine, and “b” indicates that the inhibitory response to guanosine is significantly greater in the presence of adenosine.

A similar, although even more striking, interaction between guanosine and adenosine on [^3^H]-thymidine incorporation was observed in vascular smooth muscle cells isolated from resistance arteries (i.e., preglomerular vascular smooth muscle cells) from normotensive WKY rats. In this regard, in the absence of adenosine, guanosine at concentrations as high as 100 μmol/L had no significant effect on [^3^H]-thymidine incorporation (Fig. 2, left panel). However, in the presence of adenosine, guanosine profoundly reduced [^3^H]-thymidine incorporation. For example, in the presence of 3 μmol/L adenosine (Fig. 2, middle panel), 100 μmol/L guanosine reduced [^3^H]-thymidine incorporation by 43 ± 2%; and in the presence of 10 μmol/L adenosine (Fig. 1, right panel), 100 μmol/L guanosine reduced [^3^H]-thymidine incorporation by 52 ± 2%. Analysis by two-factor ANOVA again indicated a highly significant (*P* < 0.000001) interaction between guanosine and adenosine on [^3^H]-thymidine incorporation.

**Figure 3:**
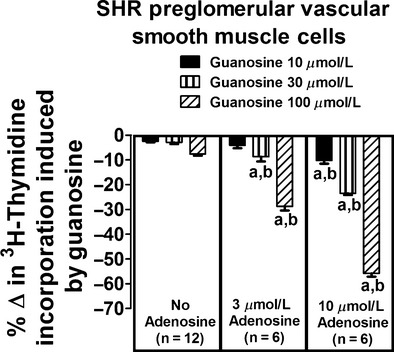
Effects of guanosine on [^3^H]-thymidine incorporation in preglomerular vascular smooth muscle cells isolated from spontaneously hypertensive rats (SHR) in the absence and presence of adenosine. Values represent means and SEMs. Two-factor ANOVA indicated a significant interaction between guanosine and adenosine on [^3^H]-thymidine incorporation (*P* < 0.000001). Letter “a” indicates a significant inhibitory response to guanosine at the indicated concentration of adenosine, and “b” indicates that the inhibitory response to guanosine is significantly greater in the presence of adenosine.

To determine whether genetic predisposition to hypertension might affect the guanosine–adenosine mechanism, we repeated the aforementioned experiments in preglomerular vascular smooth muscle cells isolated from hypertensive SHR rats. In these cells the interaction between guanosine and adenosine was also striking. In the absence of adenosine, guanosine at concentrations as high as 100 μmol/L had no significant effect on [^3^H]-thymidine incorporation (Fig. 3, left panel). However, in the presence of adenosine, guanosine profoundly reduced [^3^H]-thymidine incorporation. For example, in the presence of 3 μmol/L adenosine (Fig. 3, middle panel), 100 μmol/L guanosine reduced [^3^H]-thymidine incorporation by 29 ± 2%; and in the presence of 10 μmol/L adenosine (Fig. 1, right panel), 100 μmol/L guanosine reduced [^3^H]-thymidine incorporation by 56 ± 1%. Analysis by two-factor ANOVA again indicated a highly significant (*P* < 0.000001) interaction between guanosine and adenosine on [^3^H]-thymidine incorporation.

### Role of adenosine receptors in mediating the interaction between guanosine and adenosine on [^3^H]-thymidine incorporation in vascular smooth muscle cells

**Figure 4:**
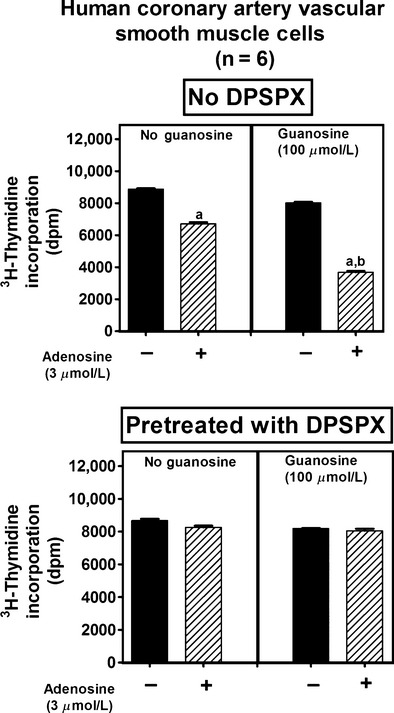
Effects of adenosine on [^3^H]-thymidine incorporation in human coronary artery vascular smooth muscle cells in the absence (left panel) and presence (right panel) of guanosine, both without (no DPSPX; top graph) and with (pretreated with DPSPX; bottom graph) 1,3-dipropyl-8-(*p*-sulfophenyl)xanthine (DPSPX; 100 μmol/L; adenosine receptor antagonist). Values represent means and SEMs. Two-factor ANOVA indicated a significant interaction between guanosine and adenosine on [^3^H]-thymidine incorporation in the “no DPSPX” group (*P* < 0.000001) but not in the “pretreated with DPSPX” group. Letter “a” indicates significantly different from corresponding group without adenosine, and “b” indicates significantly different from corresponding “no guanosine” group.

**Figure 5:**
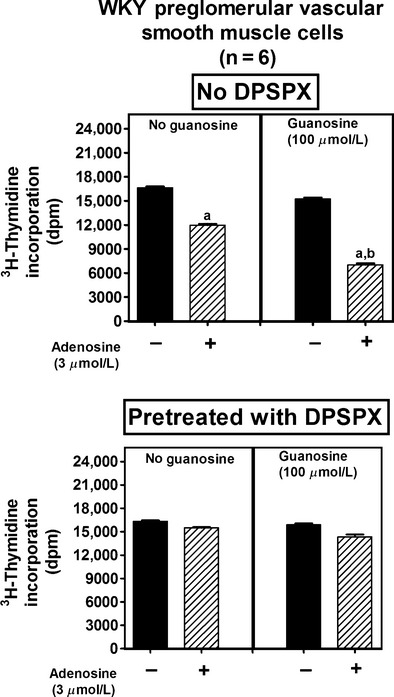
Effects of adenosine on [^3^H]-thymidine incorporation in preglomerular vascular smooth muscle cells isolated from normotensive Wistar-Kyoto rats (WKY) in the absence (left panel) and presence (right panel) of guanosine, both without (no DPSPX; top graph) and with (pretreated with DPSPX; bottom graph) 1,3-dipropyl-8-(*p*-sulfophenyl)xanthine (DPSPX; 100 μmol/L; adenosine receptor antagonist). Values represent means and SEMs. Two-factor ANOVA indicated a significant interaction between guanosine and adenosine on [^3^H]-thymidine incorporation in the “no DPSPX” group (*P* < 0.000001) but not in the “pretreated with DPSPX” group. Letter “a” indicates significantly different from corresponding group without adenosine, and “b” indicates significantly different from corresponding “no guanosine” group.

**Figure 6:**
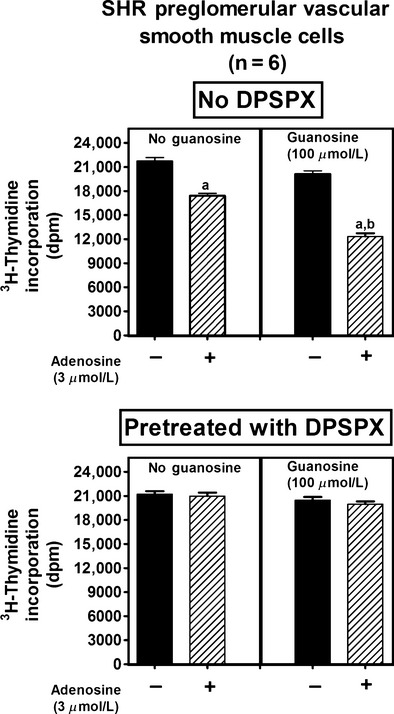
Effects of adenosine on [^3^H]-thymidine incorporation in preglomerular vascular smooth muscle cells isolated from spontaneously hypertensive rats (SHR) in the absence (left panel) and presence (right panel) of guanosine, both without (no DPSPX; top graph) and with (pretreated with DPSPX; bottom graph) 1,3-dipropyl-8-(*p*-sulfophenyl)xanthine (DPSPX; 100 μmol/L; adenosine receptor antagonist). Values represent means and SEMs. Two-factor ANOVA indicated a significant interaction between guanosine and adenosine on [^3^H]-thymidine incorporation in the “no DPSPX” group (*P* = 0.0002) but not in the “pretreated with DPSPX” group. Letter “a” indicates significantly different from corresponding group without adenosine, and “b” indicates significantly different from corresponding “no guanosine” group.

Our previous studies show that adenosine inhibits vascular smooth muscle cell proliferation via adenosine receptors (Dubey et al. 1996a,b, 1998; Jackson et al. 2011a,b) and that guanosine increases extracellular adenosine (Jackson et al. 2013). Therefore, if our hypothesis that the guanosine–adenosine mechanism regulates cell proliferation is correct, the interaction between guanosine and adenosine on [^3^H]-thymidine incorporation should be blocked by an adenosine receptor antagonist. To test this prediction, human coronary artery, WKY preglomerular, and SHR preglomerular vascular smooth muscle cells were incubated with adenosine (3 μmol/L), guanosine (100 μmol/L), or adenosine plus guanosine both in the absence and presence of DPSPX (adenosine receptor antagonist [Daly and Jacobson 1995]; 100 μmol/L). In the absence of DPSPX, we again observed a highly significant interaction between guanosine and adenosine on [^3^H]-thymidine incorporation (*P* < 0.000001, *P* < 0.000001, and *P* = 0.0002 in human coronary artery, WKY preglomerular, and SHR preglomerular vascular smooth muscle cells, respectively; top panels of Figs. 4–6, respectively). Importantly, the interaction between guanosine and adenosine on [^3^H]-thymidine incorporation was abrogated by DPSPX (*P*-value for interaction between guanosine and adenosine in the presence of DPSPX: *P* = 0.1914, *P* = 0.0920, and *P* = 0.7542 in human coronary artery, WKY preglomerular, and SHR preglomerular vascular smooth muscle cells, respectively (bottom panels of Figs. 4–6, respectively).

### Role of SLC19A1, SLC19A2, SLC19A3, and SLC22A2 in mediating the interaction between guanosine and adenosine

To confirm that extracellular guanosine does indeed increase extracellular adenosine and to explore the mechanism, we incubated SHR preglomerular vascular smooth muscle cells with adenosine (3 μmol/L) in the absence and presence of guanosine (30 μmol/L) and in the absence and presence of compounds that are archetypal substrates for either SLC19A1, SLC19A2, SLC19A3, or SLC22A2. After 1 h, the medium was collected and analyzed by mass spectrometry for adenosine. Our rationale for focusing on SLC19A1, SLC19A2, SLC19A3, and SLC22A2 is that (1) our previous studies seem to rule out the classical pathways for adenosine disposition in the guanosine–adenosine mechanism (Jackson et al. 2013); (2) methotrexate, which has a guanine-like polycyclic ring, increases extracellular adenosine levels (Chan and Cronstein 2002; Tian and Cronstein 2007) and methotrexate is transported by members of the SLC19 family of transporters (SLC19A1, SLC19A2, and SLC19A3) (Ganapathy et al. 2004); and (3) SLC22A2 can mediate the transmembrane transport of guanosine analogs (Cheng et al. 2012).

**Figure 7:**
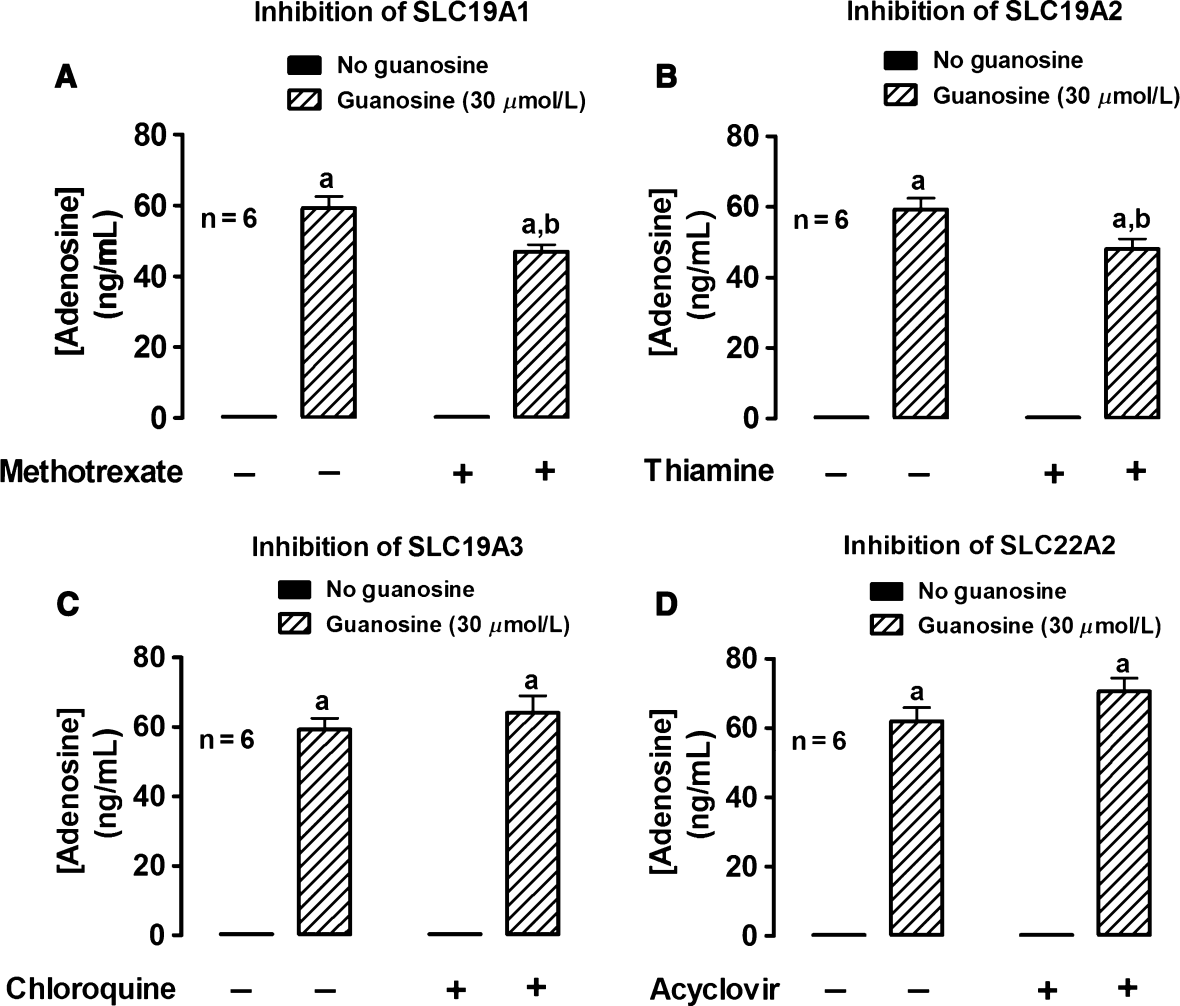
Rat preglomerular vascular smooth muscle cells isolated from spontaneously hypertensive rats were incubated with adenosine (3 μmol/L) for 1 h in the absence or presence of guanosine (30 μmol/L) and either without or with: (A) methotrexate (0.1 mmol/L; SLC19A1 inhibitor); (B) thiamine (1 mmol/L; SLC19A2 inhibitor); (C) chloroquine (1 mmol/L; SLC19A3 inhibitor); or (D) acyclovir (10 mmol/L; SLC22A2 inhibitor). The medium was assayed for adenosine by mass spectrometry. Values represent means and SEMs. Two-factor ANOVA indicated a significant interaction between methotrexate and guanosine (*P* = 0.0038) and thiamine and guanosine (*P* = 0.0175), but not between chloroquine and guanosine or acyclovir and guanosine. Letter “a” indicates significantly different from corresponding “no guanosine” group, “b” indicates significantly different from corresponding “guanosine” group.

As shown in Figure 7 and as observed previously (Jackson et al. 2013), after 1 h of incubating 3 μmol/L of adenosine with preglomerular vascular smooth muscle cells, adenosine in the medium was nearly below the detection limit of our assay system. In contrast, in the presence of guanosine, adenosine levels in the medium remained elevated even after 1 h of incubation with the cells. Very high concentrations of methotrexate (substrate for SLC19A1 [Ganapathy et al. 2004]), thiamine (substrate for SLC19A2 [Ganapathy et al. 2004]), chloroquine (substrate for SLC19A3 [Ganapathy et al. 2004]), and acyclovir (substrate for SLC22A2 [Cheng et al. 2012]) did not alter the rapid disposition of extracellular adenosine. Moreover, guanosine still attenuated adenosine disposition even in the presence of these compounds.

## Discussion

Adenosine is an autocrine and paracrine factor that exerts a wide spectrum of actions by activating A_1_, A_2A_, A_2B_, or A_3_ adenosine receptors (Jacobson 2009; Fredholm 2010; Trincavelli et al. 2010). Via these receptors, adenosine regulates multiple organ systems including the kidneys (Vallon et al. 2006), heart (Mustafa et al. 2009), liver (Peng et al. 2008), brain (Sebastiao and Ribeiro 2009), lungs (Mohsenin and Blackburn 2006; Vass and Horvath 2008), bladder (Yu et al. 2006, 2011), skeletal muscle (Hespel and Richter 1998; Marshall 2002), adipose tissue (Fredholm et al. 2011), autonomic nervous system (Westfall et al. 1990; Pelleg et al. 1997), and immune system (Bours et al. 2006; Kumar and Sharma 2009). Moreover, adenosine protects against ischemia–reperfusion injury (Eltzschig 2009), modulates fibrosis (Chan and Cronstein 2010), and facilitates wound healing (Feoktistov et al. 2009; Valls et al. 2009). Because adenosine is produced in the extracellular compartment via ectoenzymes, newly formed adenosine is well positioned to modulate cell function by stimulating its cell-surface receptors.

Steady state levels of extracellular adenosine depend not only on the rate of extracellular production of adenosine, but also on the rate of disposition of adenosine from the extracellular compartment (Grenz et al. 2012). In this regard, because of highly efficient disposition mechanisms, extracellular adenosine has an ultra-short half-life of seconds to minutes in the extracellular compartment (Eltzschig 2009). For this reason, inhibitors of the disposition of adenosine can substantially increase extracellular levels of adenosine (Eltzschig 2009). Our previous study shows that extracellular guanosine is a potential endogenous regulator of adenosine disposition from the extracellular compartment (Jackson et al. 2013). But whether this “guanosine–adenosine mechanism” has functional consequences is an open question.

The results of the present study are consistent with the concept that the guanosine–adenosine mechanism indeed entails functional consequences. In this regard, here we show that guanosine interacts with adenosine in such a manner that the ability of the combination to inhibit proliferation of vascular smooth muscle cells is greater than the sum of their effects individually, that is, there is a highly statistically significant interaction between guanosine and adenosine on cell proliferation. Because our previous work shows that endogenous extracellular levels of adenosine and guanosine are associated and that blocking increases in endogenous extracellular guanosine attenuates increases in endogenous extracellular adenosine (Jackson et al. 2013), it is likely that the guanosine–adenosine mechanism is an endogenous system that regulates vascular smooth cell proliferation.

The current studies confirm that the interaction between guanosine and adenosine on cell proliferation occurs in vascular smooth muscle cells from both coronary arteries and preglomerular microvessels and vascular smooth muscle cells from both normotensive and genetically hypertensive animals. The finding of similar results in all three cell types indicates the importance of the mechanism and the reproducibility of the findings. These findings are also important because overproliferation of vascular smooth muscle cells is involved in diseases of both large conduit arteries (e.g., neointimal hyperplasia and restenosis following stenting of coronary arteries with bare metal stents [Curcio et al. 2011]) and small microvessels (for example, microvascular remodeling associated with hypertension [Dubey et al. 1997]). Therefore, it is conceivable that the guanosine–adenosine mechanism could be involved in protecting both large and small arteries from pathological changes. It is also possible that this mechanism could be exploited as a therapy to prevent/treat inappropriate remodeling of large and small arteries.

Because microvascular hyperplasia may contribute to the vascular pathology in genetic hypertension (Campbell et al. 1991) and because the guanosine–adenosine mechanism appears to attenuate microvascular smooth muscle cell proliferation, in the present study we also determined whether the guanosine–adenosine mechanism is operative in vascular smooth muscle cells isolated from the preglomerular microcirculation of SHR. The present results support the conclusion that even in the setting of genetic hypertension, the guanosine–adenosine mechanism is functional. This fact means that the mechanism could be manipulated to benefit microvascular health in hypertensive patients.

We interpret the fact that guanosine enhances adenosine-induced inhibition of cell proliferation as evidence for a functional guanosine–adenosine mechanism. However, there are other interpretations of this finding. For example, guanosine may be signaling independently of indirect activation of adenosine receptors. However, the fact that guanosine per se has little, if any, effect on vascular smooth muscle cell proliferation is inconsistent with the hypothesis that guanosine is signaling independently of indirect adenosine receptor activation. Further evidence for this conclusion is the fact that blockade of adenosine receptors with DPSPX abolishes the guanosine–adenosine interaction on cell proliferation in all three cell lines used in the present study. As it is well established that adenosine inhibits vascular smooth muscle cell proliferation via activation of cell-surface receptors (Dubey et al. 1996a,b, 1998; Jackson et al. 2011a,b), the fact that DPSPX blocks the functional consequences of the guanosine–adenosine interaction is entirely consistent with the thesis that guanosine alters smooth muscle cell proliferation indirectly via elevating extracellular levels of adenosine (and hence increasing adenosine receptor activation). Finally, the observation that adenosine levels are nearly below detection limit in cells not treated with guanosine, yet are high in cells treated with guanosine is yet further evidence for the concept that guanosine exerts functional effects indirectly by elevating extracellular levels of adenosine. However, it is certainly conceivable that in addition to increasing extracellular adenosine levels, guanosine could exert allosteric effects on adenosine receptors and thereby promote adenosine-mediated signaling.

Because guanosine elevates adenosine levels and because this mechanism induces functional outcomes, it is important to establish the mechanism by which extracellular guanosine elevates extracellular adenosine. Previously, we examined the possibility that guanosine directly inhibits enzymes that metabolize extracellular adenosine or interferes with the ability of ENTs or CNTs to move adenosine into the cell (Jackson et al. 2013). Our previous findings, however, were inconsistent with the notion that guanosine inhibits adenosine deaminase, adenosine kinase, S-adenosylhomocysteine hydrolase or guanine deaminase, and also inconsistent with the concept that guanosine elevates extracellular adenosine levels by inhibiting ENTs or CNTs (Jackson et al. 2013).

The solute carrier superfamily of transporters includes over 300 isoforms that are organized into at least 52 subfamilies. ENTs are SLC29 subfamily members and CNTs are SLC28 subfamily members (Elwi et al. 2006). Although ENTs and CNTs are considered the primary carriers of nucleosides such as adenosine and guanosine (Elwi et al. 2006), there are other SLC subfamilies that may transport nucleosides. For example, methotrexate, which has a guanine-like polycyclic ring, is transported by members of the SLC19 family of transporters (SLC19A1, SLC19A2, and SLC19A3) (Ganapathy et al. 2004), and SLC22A2 can mediate the transmembrane transport of guanosine analogs (Cheng et al. 2012). It is conceivable, therefore, that these SLCs are involved in the guanosine–adenosine mechanism. However, the present study demonstrates that extremely high concentrations of drugs known to be transported by these SLCs do not mimic the effects of guanosine nor block the effects of guanosine on extracellular adenosine. Thus, the underlying molecular basis for the guanosine–adenosine mechanism remains elusive. This suggests that a novel mechanism may be involved. For example, it is possible that guanosine modulates the rate or extent of adenosine receptor internalization and desensitization, which would both reduce the rate of adenosine clearance from the extracellular compartment and augment adenosine receptor signaling. Indeed, we have previously observed that blockade of A_1_ adenosine receptors augments the levels of extracellular adenosine (Andresen et al. 1999).

In conclusion, the present experiments demonstrate that extracellular guanosine and extracellular adenosine interact to inhibit vascular smooth muscle cell proliferation. This interaction is likely mediated by the ability of extracellular guanosine to elevate extracellular levels of adenosine, thus leading to increased activation of adenosine receptors. Although the mechanism by which guanosine increases extracellular adenosine remains elusive, this interaction may afford therapeutic opportunities.
